# Diagnostic Performance of a Novel Optical Coherence Tomography–Derived Fractional Flow Reserve: A Prospective Multicenter Study

**DOI:** 10.1002/mco2.71015

**Published:** 2026-09-11

**Authors:** Junyan Jin, Delong Chen, Xuebo Liu, Hao Zhou, Xueqiang Guan, Pengcheng Li, Jiniu Huang, Chenyun Zhang, Abuduwufuer Yidilisi, Yuxuan Zhang, Jiacheng Fang, Yiyue Zheng, Qiyue Gao, Haibo Chen, Qiyuan Xu, Jianan Wang, Jun Jiang

**Affiliations:** ^1^ Department of Cardiology, The Second Affiliated Hospital, School of Medicine Zhejiang University Hangzhou China; ^2^ State Key Laboratory of Transvascular Implantation Devices Hangzhou China; ^3^ Heart Regeneration and Repair Key Laboratory of Zhejiang Province Hangzhou China; ^4^ Transvascular Implantation Devices Research Institute Hangzhou China; ^5^ Department of Cardiology Tongji Hospital Affiliated to Tongji University Shanghai China; ^6^ Department of Cardiology The First Affiliated Hospital of Wenzhou Medical University Wenzhou China; ^7^ Department of Cardiology The Second Affiliated Hospital of Wenzhou Medical University Wenzhou China

**Keywords:** fractional flow reserve, intracoronary imaging, optical coherence tomography–derived fractional flow reserve, physiology

## Abstract

Optical coherence tomography–derived fractional flow reserve (OCT‐FFR) offers a combined assessment of anatomical and physiological lesions. However, its diagnostic accuracy needs improvement. In this prospective, multicenter, single‐arm study, 210 patients across four centers with indications for percutaneous coronary intervention underwent simultaneous FFR and OCT‐FFR assessments. OCT‐FFR was computed using a novel multitask learning algorithm that integrated lumen segmentation and bifurcation detection, enabling fluid dynamics‐based modeling of pressure gradients across coronary lesions. The primary outcome was the diagnostic accuracy of OCT‐FFR. The per‐protocol analysis included 200 lesions in 200 patients. The mean stenosis diameter was 67.43 ± 9.01%, and mean FFR was 0.85 ± 0.08. Using FFR ≤ 0.80 as the reference, OCT‐FFR showed an accuracy of 98.0%, sensitivity of 93.6%, specificity of 99.3%, and positive predictive value of 97.8%. It showed strong correlation (*r* = 0.95, *p* < 0.001) and agreement (mean difference: −0.001 ± 0.026) with FFR. Furthermore, it demonstrated superior diagnostic accuracy compared with previous methods without patient‐specific blood pressure (98.0% vs. 90.0%) or minimal lumen area optimization (98.0% vs. 93.0%) (both *p* < 0.01). The new algorithm exhibits excellent diagnostic performance and strong concordance with invasive FFR, potentially optimizing revascularization strategies. However, larger prospective cohorts are needed to validate these findings and confirm generalizability.

**Trial Registration:**

ChiCTR2500098052.

## Graphical abstract

This prospective multicenter study validated a novel OCT‐derived fractional flow reserve algorithm against invasive FFR, demonstrating excellent diagnostic accuracy, strong correlation and agreement, and significantly superior performance over traditional anatomical parameters, supporting its clinical feasibility for combined anatomical‐physiological coronary assessment.

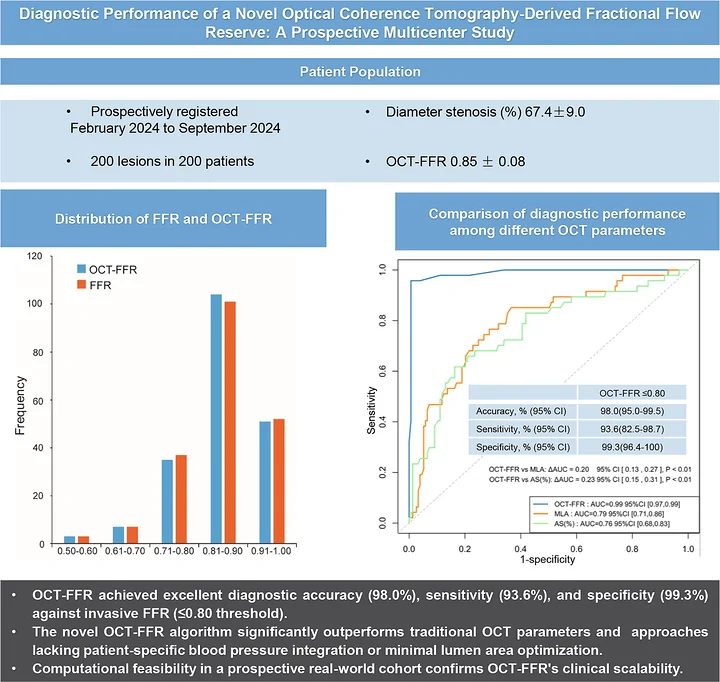

## 1 Introduction

The application of either physiological assessment or intravascular imaging reportedly optimizes percutaneous coronary intervention (PCI) procedure, improving both procedural safety and clinical outcomes [1, 2, 3]. Fractional flow reserve (FFR), the gold standard for evaluating hemodynamic significance in coronary stenosis during invasive angiography, remains central to treatment decision‐making [4]. Its clinical value has been demonstrated in patients with chronic coronary syndrome, low‐risk acute coronary syndrome, and acute myocardial infarction [5, 6, 7]. Despite strong recommendations from current guidelines, the adoption of wire‐based FFR in catheterization laboratories remains low. Such persistent underutilization highlights the urgent clinical need for a more accessible and efficient approach to physiological assessment.

Intracoronary optical coherence tomography (OCT) imaging, with its unparalleled 10–15 µm resolution, enables precise three‐dimensional reconstruction of coronary anatomy, providing critical morphologic data for computational FFR modeling [8, 9]. While OCT‐guided PCI optimization enhances clinical outcomes, concurrent intraprocedural adoption of OCT and invasive FFR is constrained by technical complexity, cost considerations, prolonged procedure times, and safety concerns. This limitation has driven the emergence of intravascular imaging‐derived physiology, a paradigm integrating morphological and functional assessment through a single modality to streamline precision‐guided PCI [10, 11].

Recent advances in computational algorithms now permit optical coherence tomography–derived fractional flow reserve (OCT‐FFR), enabling simultaneous characterization of plaque morphology and hemodynamic significance [12, 13, 14, 15]. However, current OCT‐FFR techniques demonstrate only moderate diagnostic performance and are typically time‐consuming [11].

This study aimed to prospectively evaluate the diagnostic accuracy of OCT‐FFR against that of invasive FFR in patients with coronary artery stenosis. Given that the acute phase of myocardial infarction (MI) may involve microvascular dysfunction and vasomotor instability, which could confound invasive FFR measurements [16, 17], the present study initially focused on patients with chronic coronary syndrome and unstable angina to ensure a reliable reference standard for validating the novel OCT‐FFR algorithm.

## 2 Results

### 2.1 Baseline Clinical and Lesion Characteristics

**FIGURE 1:**
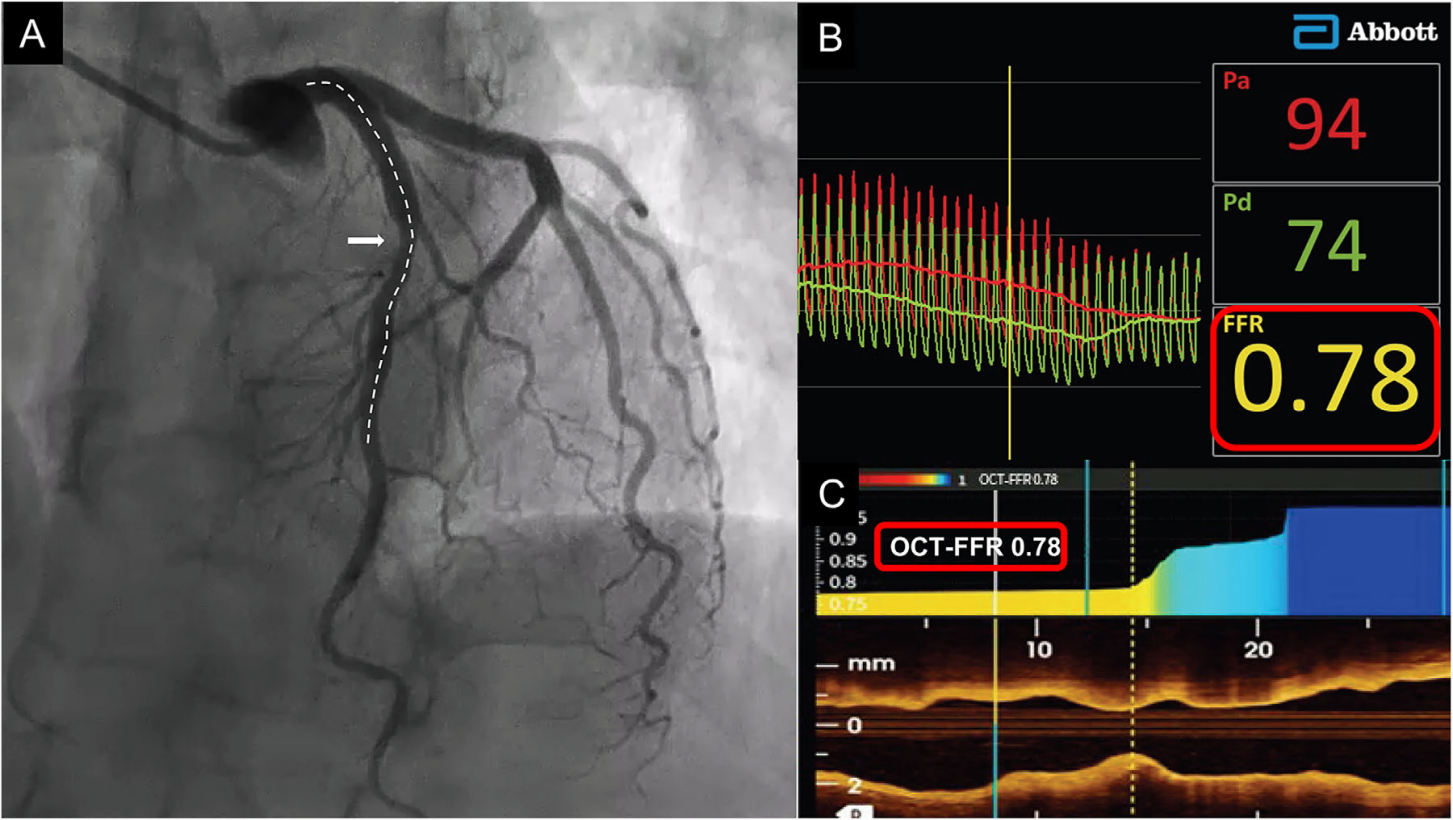
Representative example of OCT‐FFR computation in an intermediate stenosis lesion. (A) coronary angiography reveals a lesion in the left anterior descending artery. (B) FFR measured at a distal site is 0.78. (C) The computed OCT‐FFR is 0.78 at the indicated site. FFR, fractional flow reserve; OCT‐FFR, optical coherence tomography–derived FFR.

A total of 210 patients indicated for coronary angiography were screened across four Chinese centers. After excluding 10 patients, 200 patients (200 vessels) were prospectively included in the final analysis (Figure S1). Table 1 presents the baseline clinical and procedural characteristics. Of the 200 assessed vessels, 139 (69.5%) were located in the left anterior descending artery (LAD). Bifurcation lesions were present in 65 vessels (32.5%) and tandem lesions in 42 vessels (21%). The mean minimal lumen area (MLA) was 2.84 ± 1.42 mm^2^. An FFR ≤ 0.80 was observed in 47 vessels (23.5%). Figure 1 shows a representative case illustrating the OCT‐FFR computational methodology. No adverse events related to OCT‐FFR were observed during the study.

**TABLE 1:** Baseline characteristics.

	Patients (*n* = 200)
Clinical	
Age, years	66.70 ± 8.61
Male	67.0 (134)
Body mass index, kg/m^2^	24.50 ± 3.07
Diabetes	32.5 (65)
Hypertension	59.0 (118)
Hyperlipidemia	22.5(45)
Current smoker	25.0(50)
Previous percutaneous coronary intervention	15.0 (30)
Clinical presentation	
Chronic coronary syndrome	82.5(165)
Unstable angina	17.5(35)
Procedural	
Lesion location	
Left anterior descending artery	69.5 (139)
Left circumflex artery	5.0 (10)
Right coronary artery	25.5 (51)
In‐stent restenosis lesions	6.5(13)
Bifurcation lesions	32.5(65)
Tandem lesions	21.0(42)
Diffuse disease	37.5(75)
Reference vessel diameter, mm	2.72 ± 0.40
Diameter stenosis	67.43 ± 9.01
MLA of target lesion, mm^2^	2.84 ± 1.42
Mean reference segment lumen diameter, mm	3.16 ± 0.56
Minimal lumen diameter of target lesion, mm	1.89 ± 0.45
Percent area stenosis	63.62 ± 14.86
FFR	0.85 ± 0.08
FFR ≤ 0.80	23.5(47)
0.75 ≤ FFR ≤ 0.85	37.0(74)
OCT‐FFR	0.85 ± 0.08
OCT‐FFR ≤ 0.80	22.5(45)
0.75 ≤ OCT‐FFR ≤ 0.85	29.5(59)

*Note*: Values are mean ± SD or % (*n*).

Abbreviations: MLA, minimal lumen area; FFR, fractional flow reserve; OCT‐FFR, optical coherence tomography–derived fractional flow reserve.

### 2.2 Agreement Between Oct‐FFR and Invasive FFR

**FIGURE 2:**
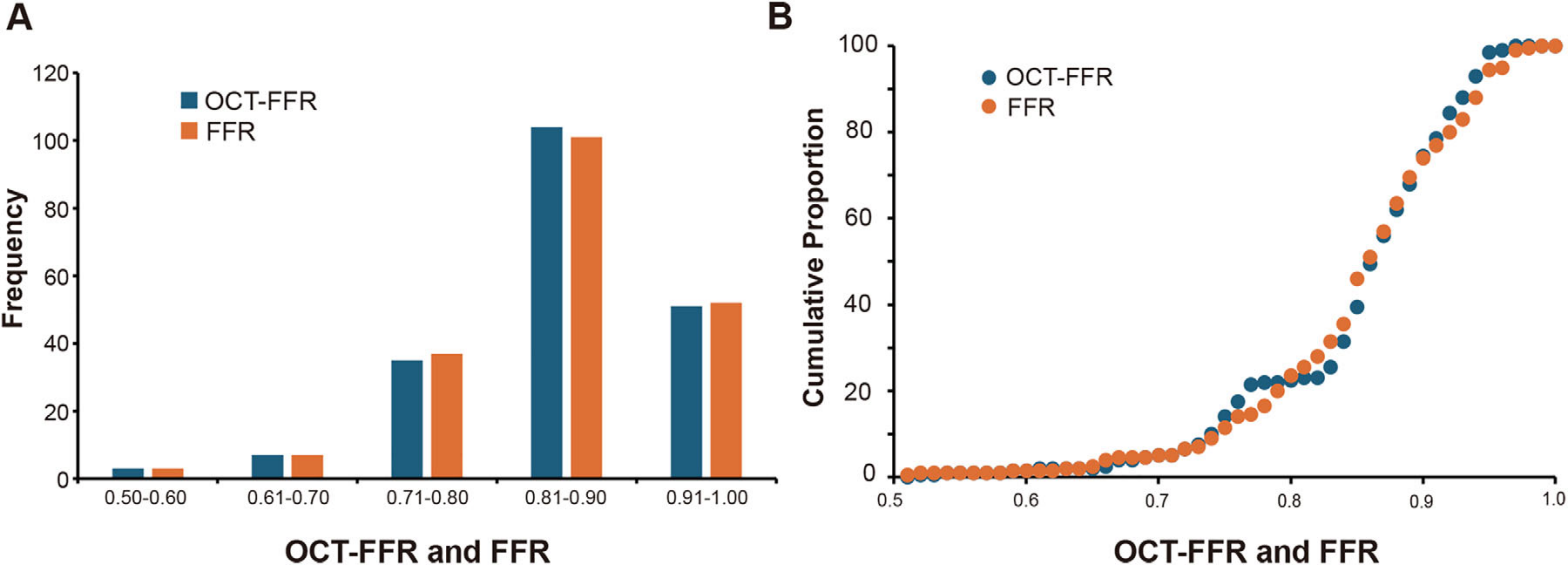
Distribution of FFR and OCT‐FFR. (A) Histogram and (B) cumulative frequency distribution curves of FFR and OCT‐FFR. FFR, fractional flow reserve; OCT‐FFR, optical coherence tomography–derived FFR.

**FIGURE 3:**
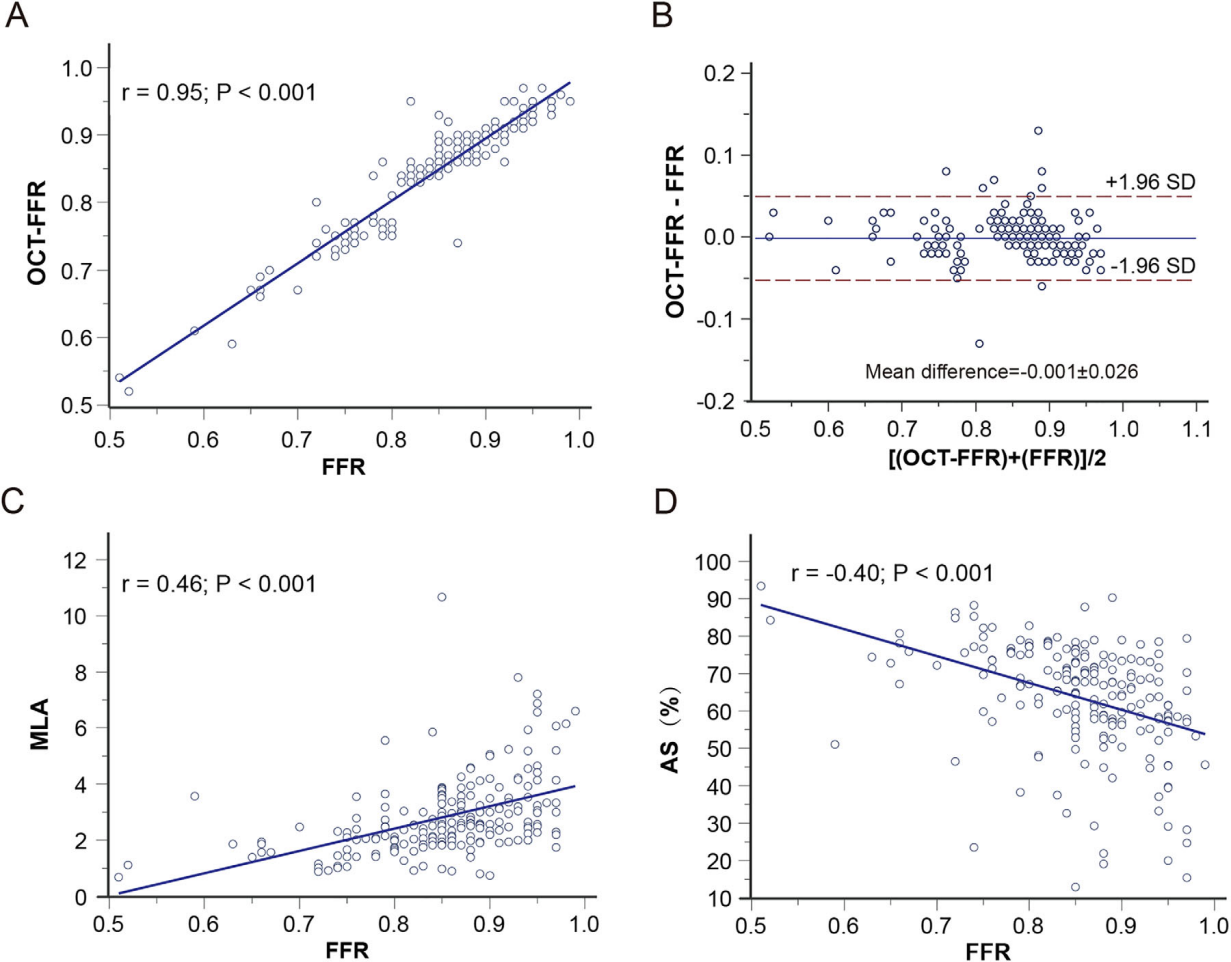
Correlation and agreement for computational FFR and wire‐based FFR. (A) Correlation between OCT‐FFR and FFR. (B) Agreement between OCT‐FFR and FFR. C() Correlation between MLA and FFR. (D) Correlation between AS and FFR. AS, area stenosis; FFR, fractional flow reserve; MLA, minimal lumen area; OCT‐FFR, optical coherence tomography–derived FFR.

Both invasive FFR and OCT‐FFR demonstrated comparable mean values (0.85 ± 0.08 each). Figure 2 shows the distribution profiles of the FFR and OCT‐FFR measurements. A strong correlation was observed between OCT‐FFR and invasive FFR (Pearson's correlation coefficient: *r* = 0.95, *p* < 0.001), with excellent agreement evidenced by Bland–Altman analysis (mean difference: −0.001 ± 0.026, *p* < 0.001; Figure 3). In contrast, OCT‐derived anatomical parameters exhibited only moderate correlations with invasive FFR.

### 2.3 Diagnostic Performance of OCT‐Derived Parameters

Using an invasive FFR threshold of ≤ 0.80 to define hemodynamically significant stenosis, OCT‐FFR demonstrated robust diagnostic performance: accuracy 98.0% (95% confidence interval [CI]: 95.0–99.5), sensitivity 93.6% (82.5–98.7), specificity 99.3% (96.4–100), positive predictive value (PPV) 97.8% (86.2–99.7), and negative predictive value (NPV) 98.1% (94.4–99.3) (Table 2). The lower bound of the CI for accuracy (95.0%) exceeded the prespecified performance goal of 84% (*p* < 0.001). OCT‐FFR maintained high diagnostic performance across lesions with borderline FFR values (0.75–0.85), intermediate stenosis, LAD, and non‐LAD locations, regardless of lesion complexity, plaque morphology, or clinical characteristics including diabetes, hypertension, and smoking status (Table 2 and Tables S1–S3).

**TABLE 2:** Diagnostic performance of OCT‐FFR to identify fractional flow reserve ≤ 0.80.

	OCT‐FFR ≤ 0.80 (*n* = 200)	OCT‐FFR ≤ 0.80 in borderline FFR (0.75–0.85) (*n* = 74)	OCT‐FFR ≤ 0.80 in intermediate stenosis (40%–70%) (*n* = 149)
Accuracy, % (95% CI)	98.0 (95.0–99.5)	95.9 (88.6–99.2)	99.3 (96.3–100)
Sensitivity, % (95% CI)	93.6 (82.5–98.7)	89.7 (72.6–97.8)	100 (83.9–100)
Specificity, % (95% CI)	99.3 (96.4–100)	100 (92.1–100)	99.2 (95.7–100)
PPV, % (95% CI)	97.8 (86.2–99.7)	100 (86.8–100)	95.5 (74.9–99.3)
NPV, % (95% CI)	98.1 (94.4–99.3)	93.8 (88.6–99.2)	100 (97.1–100)

*Note*: Values are % (95% CI). OCT‐FFR represents our novel OCT‐FFR algorithm.

Abbreviations: OCT‐FFR, optical coherence tomography–derived fractional flow reserve; CI, confidence interval; PPV, positive predictive value; NPV, negative predictive value.

**FIGURE 4:**
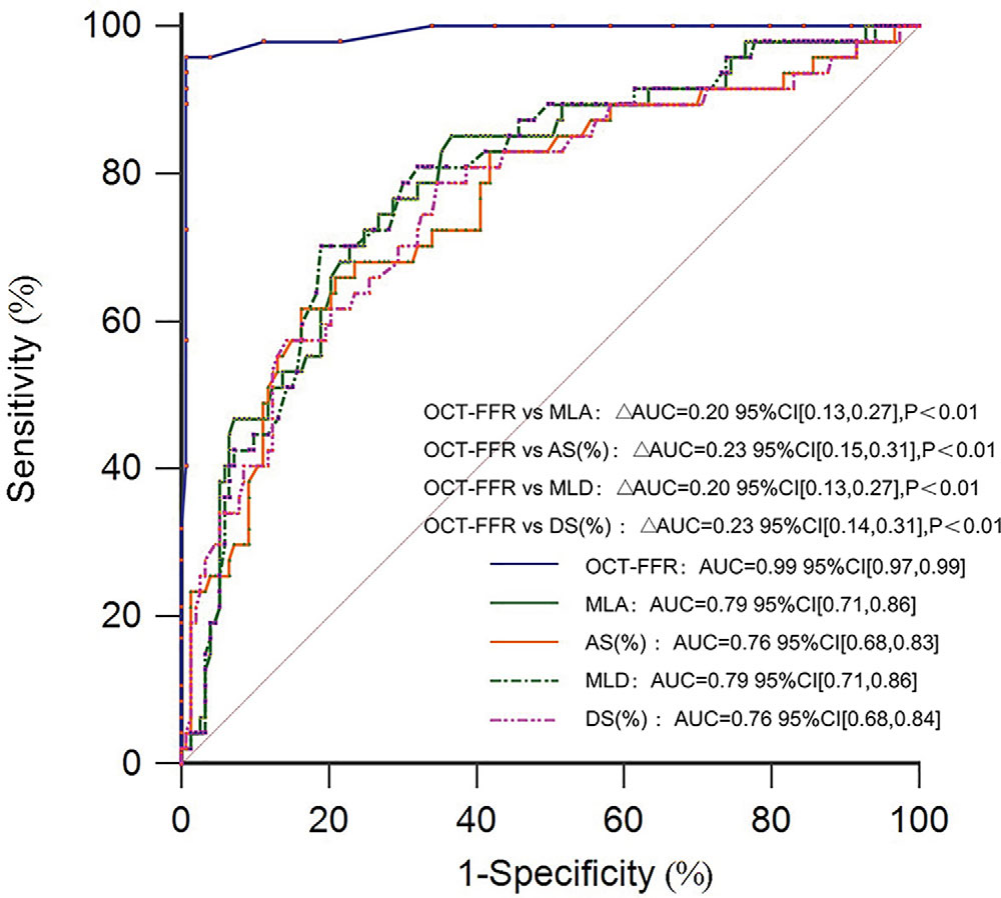
Comparison of diagnostic performance for OCT‐FFR, OCT‐derived MLA, OCT‐derived MLD, OCT‐derived DS, and OCT‐derived AS. AS, area stenosis; DS, diameter stenosis; MLA, minimal lumen area; MLD, minimal lumen diameter; OCT‐FFR, optical coherence tomography–derived FFR.

In addition, the novel OCT‐FFR algorithm demonstrated significantly superior diagnostic accuracy (98.0%) compared with methods without patient‐specific blood pressure (90.0%) and those lacking MLA optimization (93.0%) (both *p* < 0.01) (Table 3). Receiver operating characteristic (ROC) curve analysis also revealed superior discriminatory capacity for OCT‐FFR (area under curve [AUC]: 0.99, 95% CI: 0.97–0.99) compared to OCT‐derived anatomical parameters, including MLA (AUC: 0.79, 95% CI: 0.71–0.86), minimal lumen diameter (MLD) (AUC: 0.79, 95% CI: 0.71–0.86), % diameter stenosis (DS) (AUC: 0.76, 95% CI: 0.68–0.84), and % area stenosis (AS) (AUC: 0.76, 95% CI: 0.68–0.83) (Figure 4).

**TABLE 3:** Comparing of different OCT‐FFR calculating algorithms.

	OCT‐FFR ≤ 0.80	OCT‐FFR_MAP_ ≤ 0.80	OCT‐FFR_MLA_ ≤ 0.80	*P* (OCT‐FFR vs. OCT‐FFR_MAP_)	*P* (OCT‐FFR vs. OCT‐FFR_MLA_)
Accuracy, % (95% CI)	98.0 (95.0–99.5)	90.0 (85.0–93.8)	93.0 (88.5–96.1)	< 0.01	< 0.01
Sensitivity, % (95% CI)	93.6 (82.5–98.7)	97.9 (88.7–99.9)	72.3 (57.4–84.4)	0.31	< 0.01
Specificity, % (95% CI)	99.3 (96.4–100)	87.6 (81.2–92.4)	99.3 (96.4–100)	< 0.01	1.00
PPV, % (95% CI)	97.8 (86.2–99.7)	70.8 (61.3–78.7)	97.1 (82.7–99.6)	< 0.01	0.82
NPV, % (95% CI)	98.1 (94.4–99.3)	99.3 (95.1–99.9)	92.1 (88.0–94.9)	0.40	< 0.01

*Note*: OCT‐FFR represents our finalized algorithm; OCT‐FFR_MAP_ denotes OCT‐FFR computed under the assumption of a uniform mean arterial pressure of 100 mmHg for every patient, rather than the individual, procedure‐specific pressure; OCT‐FFR_MLA_ follows the algorithm reported by Seike et al. [25], which uses the minimum MLA during the global OCT pullback.

Abbreviations: NPV, negative predictive value; OCT‐FFR, optical coherence tomography–derived fractional flow reserve; PPV, positive predictive value.

**FIGURE 5:**
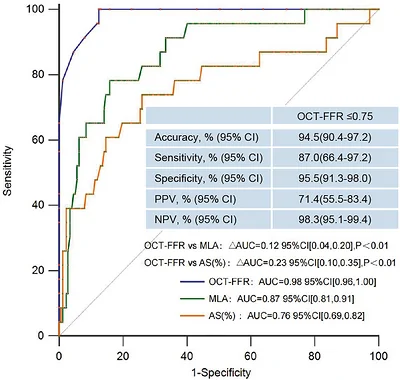
Diagnostic performance of OCT‐FFR using an invasive FFR cut‐off of ≤ 0.75 for hemodynamically significant stenosis. AS, area stenosis; MLA, minimal lumen area; OCT‐FFR, optical coherence tomography–derived FFR.

In the sensitivity analysis applying an invasive FFR cut‐off value of ≤ 0.75 to identify hemodynamically significant stenosis, a threshold of particular clinical relevance for borderline lesions where functional assessment is most challenging, OCT‐FFR continued to exhibit high diagnostic accuracy: accuracy 94.5% (95% CI: 90.4–97.2), sensitivity 87.0% (95% CI: 66.4–97.2), specificity 95.5% (95% CI: 91.3–98.0), positive predictive value (PPV) 71.4% (95% CI: 55.5–83.4), and negative predictive value (NPV) 98.3% (95% CI: 95.1–99.4). Furthermore, ROC curve analysis confirmed the superior discriminative ability of OCT‐FFR (AUC: 0.98, 95% CI: 0.96–1.00) over OCT‐based anatomical measures such as MLA (AUC: 0.87, 95% CI: 0.81–0.91) and %AS (AUC: 0.76, 95% CI: 0.69–0.82) (Figure 5). These findings highlight the robust performance of OCT‐FFR in clinically relevant borderline lesion subsets.

## 3 Discussion

This multicenter prospective study validated a novel OCT‐FFR algorithm against invasive FFR, yielding three pivotal findings. First, OCT‐FFR demonstrated strong correlation and excellent agreement with invasive FFR. Second, using an FFR ≤ 0.80 as threshold for hemodynamically significant stenosis, OCT‐FFR achieved robust diagnostic accuracy (98.0%), sensitivity (93.6%), and specificity (99.3%). Its performance remained consistent across different lesion types, with significantly superior accuracy compared to methods lacking patient‐specific blood pressure integration or MLA optimization. Third, the computational feasibility of OCT‐FFR in a real‐world prospective cohort underscores its clinical scalability. These results establish OCT‐FFR as a transformative modality that integrates micron‐scale anatomical precision with ischemia‐specific hemodynamic assessment, potentially optimizing PCI procedural planning.

OCT provides superior depth‐resolved tissue characterization and precise lumen contour delineation, enabling meticulous stent optimization. While intracoronary imaging‐ or FFR‐guided revascularization demonstrates improved clinical outcomes compared to revascularization under angiographic guidance alone, conventional intracoronary imaging remains fundamentally anatomical in nature and lacks functional assessment capabilities [18]. Although MLA measurements from intravascular imaging have historically served as surrogates for functional evaluation in FFR‐absent scenarios, their diagnostic accuracy is inconsistent, particularly in intermediate‐grade stenosis [19]. Moreover, the widespread adoption of invasive FFR faces practical constraints including prolonged procedure time, risks associated with pressure wire manipulation, and elevated procedural costs. To address this anatomy‐physiology dichotomy, computational FFR estimation techniques that utilize angiography or intravascular imaging data (intravascular ultrasound [IVUS]/OCT) to derive virtual hemodynamic indices without hyperemic stimulation have emerged. A meta‐analysis by Takahashi et al. substantiates the clinical validity of imaging‐derived computational FFR across modalities, demonstrating consistent diagnostic performance supportive of real‐world implementation [11]. Within this technological landscape, OCT‐FFR distinguishes itself through enhanced diagnostic precision, computational efficiency, and seamless integration of anatomical‐physiological data, offering clinicians a multidimensional lesion assessment tool while obviating pressure wire requirements. Despite these advancements, current evidence for imaging‐derived physiological indices remains predominantly retrospective and inherently vulnerable to selection bias and confounding factors. Early prospective validation includes a multicenter OCT‐FFR study with a relatively small sample size reporting 92% sensitivity and 93% specificity [20], while the recent FUSION trial demonstrated 80.4% sensitivity and 82.9% specificity for ischemia detection, achieving computational integration within 1 s of standard OCT acquisition [13]. Moreover, a recent substudy of the ILUMIEN IV trial prospectively demonstrated that post‐PCI OCT‑based virtual flow reserve independently predicted 2‑year clinical outcomes, further supporting the prognostic value of OCT‑derived physiology beyond anatomical metrics [21]. Parallel developments include a real‐time IVUS‐based FFR system by Okubo et al., enabling simultaneous treatment decision‐making and stent sizing through single‐pullback acquisition [10]. Our large‐scale prospective multicenter investigation further corroborates these findings, achieving 98.0% diagnostic accuracy (93.6% sensitivity, 99.3% specificity), substantiating the high clinical reliability of OCT‐FFR for functional lesion assessment.

FFR enables ischemia‐specific lesion identification through invasive pressure measurements [22, 23]. However, in the acute phase of acute coronary syndrome, transient physiological instability is frequently compounded by vasospasm and may compromise the accuracy of FFR assessment for non‐culprit lesions, potentially leading to overestimation of stenosis severity [24]. Given these potential confounders in acute settings, patients with acute MI were excluded from the present study to ensure reliability of FFR as the reference standard. Consequently, the findings of this study are primarily applicable to patients with chronic coronary syndrome or unstable angina and future studies are needed to validate OCT‐FFR in acute settings. In contrast to conventional FFR, which requires adenosine‐induced maximal hyperemia, OCT‐FFR utilizes quantitative lumen geometry from standard OCT acquisitions to compute virtual hemodynamic parameters. This innovative approach eliminates pharmacological stimulation, thereby reducing procedural time, patient discomfort, and adenosine‐related complications [25]. Notably, OCT‐FFR demonstrates particular clinical utility in addressing diagnostic uncertainties within the borderline FFR values (0.75–0.85). Our findings revealed modest diagnostic for performance in lesions within this region. While lesions exceeding FFR 0.80 may still present high‐risk plaque characteristics, those below this threshold might not universally derive benefits from revascularization. This underscores the importance of multimodal decision‐making in managing borderline lesions. In addition, OCT‐FFR facilitates comprehensive lesion assessment by integrating functional data with detailed morphological information regarding plaque composition, burden distribution, and lesion geometry [26]. Clinicians can subsequently integrate these parameters with clinical presentation and procedural complexity to guide personalized revascularization strategies and effectively resolve anatomy‐function discrepancies through precision intervention planning.

Processing speed is a pivotal determinant of technological adoption in the clinical translation of computational physiology. Although our OCT‐FFR analysis was performed in a centralized core laboratory setting that may differ from catheterization laboratory workflows, the complete computational workflow encompassing lumen segmentation, bifurcation recognition using fractal‐law‐based branch correction, three‐dimensional reconstruction, and hemodynamic simulation with real‐time blood pressure integration could be completed within 30 s per vessel. This processing speed compares favorably with existing intracoronary imaging‐based FFR computation systems, demonstrating minimal interference compared with routine interventional workflows. Moreover, we achieved a notably superior diagnostic performance with invasive FFR, surpassing the efficacy of prior algorithms. This improvement can be attributed to three critical methodological advancements: First, the dynamic integration of intraprocedural blood pressure measurements circumvents potential inaccuracies arising from fixed‐mean arterial pressure models. Second, applying the bifurcation model for branch lumen area correction reduces the cross‐sectional area measurement variability at bifurcation sites, thereby improving stenosis grading accuracy [14, 27]. Third, our fluid dynamics framework redefines the assessment of flow separation‐induced pressure loss by strategically localizing the MLA to the maximal stenosis region rather than adopting the global OCT pullback minimum. This adjustment addresses a critical physiological consideration: given the natural proximal‐to‐distal vessel tapering, the absolute MLA location may not correspond to the hemodynamically significant stenosis. Conventional MLA‐based approaches risk misidentifying distal vessel segments as critical stenosis sites, consequently inducing systematic FFR overestimation [25]. Collectively, these synergistic refinements establish OCT‐FFR as an expeditious as well as physiologically precise guidance tool for coronary interventions.

Our study has certain limitations. First, the current computational fluid dynamics models underlying OCT‐FFR calculations depend on simplified assumptions of uniform vascular resistance, neglecting localized biomechanical effects, such as calcification‐induced vascular stiffening and lipid‐related microvascular dysfunction. These factors critically influence shear stress distribution and are not fully considered for in existing algorithms. Furthermore, the generalizability of OCT‐FFR remains constrained by the lack of stratified analyses that correlate specific plaque morphologies with hemodynamic discrepancies. Future model refinements should prioritize the integration of shear stress mapping and microcirculatory resistance parameters to enhance physiological accuracy, particularly in populations with inherent microvascular abnormalities. Second, although our trial validated OCT‐FFR in non‐complex lesions, its clinical superiority may be more evident in anatomically challenging scenarios. We also excluded patients with acute MI to minimize confounding from acute‐phase physiological instability, which limits the applicability of our findings to the acute setting. In addition, lesions involving the left main or ostial location, those with myocardial bridging, and those with small vessels (< 2.0 mm) were excluded because of technical challenges in image acquisition and computational reliability, which further narrows the generalizability of our results. Notably, 25% of FFR‐negative diabetic lesions demonstrate OCT‐detected high‐risk thin‐cap fibroatheroma, a feature independently associated with adverse cardiovascular outcomes [26]. This underscores the need for prospective validation in complex coronary subsets including multivessel disease, post‐MI territories, and in‐stent restenosis where conventional functional assessment faces technical constraints. Third, persistent technical limitations affect the anatomical applicability of OCT‐FFR. Severe vessel tortuosity and circumferential calcification increase imaging artifact susceptibility, compromising lumen contour accuracy and subsequent FFR computation reliability. The systematic exclusion of small‐diameter vessels (< 2 mm) and distal lesions, owing resolution thresholds risks underestimates the ischemic burden in clinically relevant territories. Emerging technical solutions, including miniaturized imaging catheters, artificial‐intelligence‐driven artifact correction, and vessel‐specific hemodynamic criteria, may address these constraints. Hybrid approaches combining OCT‐FFR with IVUS could further enhance lesion characterization, although device compatibility requires optimization.

## 4 Conclusions

The novel OCT‐FFR algorithm demonstrated excellent diagnostic performance and strong concordance with invasive FFR for functional coronary artery evaluation, offering a novel platform for simultaneous anatomical and physiological assessment of coronary lesions. This integrated approach may enhance procedural decision‐making and potentially improve PCI outcomes. However, robust clinical evidence validating the real‐world benefits of this combined anatomical and physiological strategy remains to be established.

## 5 Materials and Methods

### 5.1 Study Design and Population

This prospective, single‐arm, multicenter observational study was conducted between February 27, 2024, and September 19, 2024. Patients were enrolled from four hospitals in China: the Second Affiliated Hospital of Zhejiang University School of Medicine, Tongji Hospital affiliated to Tongji University, the First Affiliated Hospital of Wenzhou Medical University, and the Second Affiliated Hospital of Wenzhou Medical University. Consecutive patients with chronic or acute coronary syndrome who underwent coronary angiography and had visually assessed stenosis (30%–90%) were screened. The inclusion criteria were as follows: (1) age between 18 and 80 years; (2) only one target lesion per patient, with a diameter of 2.0–3.5 mm; and (3) acquisition of clear images throughout the entire target coronary artery. The exclusion criteria were as follows: (1) acute coronary syndrome in the past 72 h, (2) persistent coronary spasm or myocardial bridge in the target vessel, (3) lesions prepared before FFR or OCT measurement, and (4) lesions located in the ostial coronary artery, left main coronary artery, or coronary artery bypass graft.

Written informed consent was obtained from all included patients. The study protocol was approved by the Institutional Ethics Committees of the Second Affiliated Hospital of Zhejiang University School of Medicine, Tongji Hospital affiliated with Tongji University, the First Affiliated Hospital of Wenzhou Medical University, and the Second Affiliated Hospital of Wenzhou Medical University. The study was performed in accordance with the Declaration of Helsinki and Good Clinical Practice and registered at https://www.chictr.org.cn (ChiCTR2500098052).

### 5.2 Study Procedures

Coronary angiography was performed using standardized multi‐projection imaging protocols. FFR assessment was performed using a pressure‐sensing guidewire (Abbott Vascular, USA) positioned distal to the target lesion. FFR values were derived from the Pd/Pa ratio, where Pd denotes mean coronary pressure measured ≥ 10 mm beyond the distal edge of the stenosed segment, while Pa represents aortic pressure measured during the exit of the guide catheter [23]. Following pharmacological vasodilation, the pressure wire was systematically withdrawn from the distal coronary segment to the ostium under continuous pressure recording. The measured value of Pd/Pa represented the FFR of the entire coronary vessel rather than the isolated angiographic narrowing segment. Lesions with FFR ≤ 0.80 were considered hemodynamically significant and recommended for revascularization.

OCT was performed using the Cornaris P80 (Vivolight, China) or Mobile OCT system (Vivolight, China) with standardized acquisition parameters: 200 frames/s imaging frequency and 40 mm/s automated pullback for P80; 200 frames/s imaging frequency and 37.5 mm/s automated pullback for Mobile. Contrast flushing protocols were optimized based on the vessel anatomy: left coronary artery injections (14 mL at 4 mL/s) and right coronary artery injections (12 mL at 3 mL/s). Before the initiating pullback, careful angiographic confirmation of the distal starting point (within 80 mm of the ostium for P80 or 75 mm for Mobile) was required to ensure complete lesion coverage of the lesion. Real‐time angiographic coregistration was employed during the PCI procedure to maintain spatial orientation.

FFR measurements and OCT imaging were performed for the target coronary vessels in a randomized sequence. When OCT preceded functional evaluation, the radio‐opaque marker of imaging catheter was positioned ≥ 10 mm distal to the target lesion using predefined angiographic landmarks. Subsequent pressure wire placement was guided by fluoroscopic alignment with the recorded position of the OCT marker. Reverse sequencing was performed according to the analogous registration principles. To ensure standardization across sites, all operators underwent centralized training on OCT acquisition protocols. The OCT imaging data were digitally stored and analyzed by an independent core laboratory (CCRC Medtech (Shanghai) Co., Shanghai). All OCT data were subjected to quality review and analysis of the segment lumen area and diameter.

### 5.3 OCT‐Derived FFR

We developed a novel OCT‐FFR algorithm based on fluid dynamic modeling of pressure gradients along coronary lesions. High‐resolution OCT imaging data enabled the reconstruction of three‐dimensional lumen geometry, with automated detection of the reference lumen area and MLA, using a multi‐task learning architecture integrating lumen segmentation and bifurcation detection. Pressure loss (Δ*P*) across stenoses was computed via:

$$\Delta P=FV+SV^{2}$$

where *F* and *S* are the pressure loss coefficients due to viscous friction and flow separation, respectively, and *V* is the flow velocity [28]. *F* and *S* could be calculated as follows:

$$F=\frac{8\pi \mu L}{A_{s}}\frac{A_{n}}{A_{s}},S=\frac{\rho }{2}{\left(\frac{A_{n}}{A_{s}}-1\right)}^{2}$$

where *μ* = 4.0 × 10^−^^3^ Pa s (blood viscosity), *ρ* = 1050 kg/m^3^ (blood density), *L* indicates lesion length, quantified as the physiologically effective vessel length after excluding the guiding catheter on the OCT pullback, *A_n_* is the reference lumen area from the proximal and distal reference frames, and *A_s_* represents the lumen area in the location of the largest stenosis rate. The total Poiseuille resistance was calculated by summing the frame‐wise resistances (200 µm longitudinal resolution). The coefficient *S* was calculated using the larger area from the proximal and distal reference frames alongside the largest stenosis rate. The maximum blood flow velocity (*V*) was determined by multiplying the baseline coronary blood flow velocity by the stenosis flow reserve (SFR) value, where SFR is established by plotting the relationship between distal coronary stenosis pressure (mean arterial pressure—Δ*P*) and relative coronary blood flow [25]. Systemic blood pressure inputs were integrated to compute translesional hemodynamics. Following the standard OCT pullback procedure, lumen and bifurcation identification and 3D reconstruction were accomplished within approximately 20 s. After incorporating the blood pressure data, the OCT‐FFR value of the target lesion was obtained in 0.018 s. The automated OCT‐FFR calculation consisted of four sequential steps performed in the background: OCT image rendering, automated lumen segmentation, fluid dynamic computation, and final result display. The median processing time from completion of OCT pullback to result display was 28.07 s (24.29–34.43 s). Notably, the OCT‐FFR calculations in this study were performed offline by an independent core laboratory blinded to the invasive FFR results and were not used to guide intraprocedural revascularization decisions. Further technical details of the OCT‐FFR algorithm, including lumen segmentation, fluid dynamics modeling, and result visualization, are provided in the Technical Appendix for OCT‐FFR in the Supporting Information.

### 5.4 Outcomes

The primary outcome was the diagnostic accuracy of OCT‐FFR (≤ 0.8 or > 0.8) to identify hemodynamically significant coronary stenosis with FFR (≤ 0.8 or > 0.8) as the reference standard. The secondary outcomes included the agreement, correlations, sensitivity, specificity, PPV, NPV, ROC, and AUC of OCT‐FFR compared with those of FFR.

### 5.5 Statistical Analyses

For the primary endpoint, a one‐sided hypothesis test was employed to determine whether the diagnostic accuracy of OCT‐FFR exceeded the prespecified target of 84% (significance level *α* = 0.025; power = 80%). Based on prior studies and assuming a 10% attrition rate, a minimum sample size of 210 patients was required [11, 13].

Continuous variables are presented as mean ± standard deviation for normally distributed data or median (interquartile range) for non‐normally distributed data, with normality assessed using Shapiro–Wilk tests or quantile‐quantile plots. Categorical variables are reported as frequencies and percentages, analyzed using the chi‐square or Fisher's exact tests. Linear regression was used to evaluate the associations between the OCT‐derived parameters and wire‐based FFR measurements. Systematic differences were assessed using the Bland–Altman analysis. The diagnostic performance was quantified using ROC‐derived AUC values, and statistical comparisons were performed using the DeLong test. All analyses were conducted using the R version 4.3.2 (R Foundation for Statistical Computing).

## Author Contributions

Conceptualization: Jianan Wang and Jun Jiang. Methodology: Jianan Wang and Jun Jiang. Funding acquisition: Jun Jiang. Supervision: Jianan Wang and Jun Jiang. Investigation: Junyan Jin, Delong Chen, Xuebo Liu, Hao Zhou, Xueqiang Guan, Pengcheng Li, Jiniu Huang, Chenyun Zhang, Abuduwufuer Yidilisi, Yuxuan Zhang, Jiacheng Fang, Yiyue Zheng, Qiyue Gao, Haibo Chen, and Qiyuan Xu. Formal analysis: Junyan Jin and Delong Chen. Data curation: Junyan Jin and Delong Chen. writing – original draft preparation: Junyan Jin and Delong Chen. writing – review and editing: Jianan Wang and Jun Jiang. All authors have read and approved the final manuscript.

## Funding

This work was supported by the Noncommunicable Chronic Diseases‐National Science and Technology Major Project (No.2023ZD0504100), the National Natural Science Foundation of China (No.82170332), and Transvascular Implantation Devices Research Institute (KY012025005) to Prof. Jiang from the Second Affiliated Hospital of Zhejiang University School of Medicine. The other participating centers received no specific funding for this study.

## Ethics Statement

Our clinical trial was approved by the institutional ethics committees of the four participating centers: Second Affiliated Hospital of Zhejiang University (No. 2023‐599), Tongji Hospital affiliated to Tongji University (No. 2024‐030), First Affiliated Hospital of Wenzhou Medical University (No. 2024‐048), and Second Affiliated Hospital of Wenzhou Medical University (No. 2024‐Q‐04‐01). Written informed consent was obtained from all participants. Our study was conducted in accordance with the Helsinki Declaration and was approved by the ethical review board of all participated centers.

## Conflicts of Interest

The authors declare no conflicts of interest.

## Supporting information




**Supporting File 1**: mco271015‐sup‐0001‐SuppMat.docx.

## Data Availability

Patient‐level data collected for this study will not be made publicly available. Any reasonable inquiries should be sent to the corresponding author.
