# Supplementary material for: Diagnostic Performance of a Novel Optical Coherence Tomography–Derived Fractional Flow Reserve: A Prospective Multicenter Study
Source: MedComm (2020). 2026 Sep 11;7(9):e71015. doi: 10.1002/mco2.71015 (PMC13570264; doi:10.1002/mco2.71015)
Supplement: Supplementary file 1 [file MCO2-7-e71015-s001.docx]

**Diagnostic Performance of a Novel Optical Coherence Tomography-Derived Fractional Flow Reserve: A Prospective Multicenter Study**

Junyan Jin^1,2,3,4,*^, Delong Chen^1,2,3,4,*^, Xuebo Liu^5^, Hao Zhou^6^, Xueqiang Guan^7^, Pengcheng Li^1^, Jiniu Huang^1^, Chenyun Zhang^1^, Abuduwufuer Yidilisi^1,4^, Yuxuan Zhang^1^, Jiacheng Fang^1^, Yiyue Zheng^1^, Qiyue Gao^1^, Haibo Chen^1^, Qiyuan Xu^1^, Jianan Wang^1,2,3,4, †^, Jun Jiang^1,2,3,4, †^

^1^Department of Cardiology, The Second Affiliated Hospital, School of Medicine, Zhejiang University, Hangzhou, 310009, China

^2^State Key Laboratory of Transvascular Implantation Devices, Hangzhou, 310009, China

^3^Heart Regeneration and Repair Key Laboratory of Zhejiang Province, Hangzhou, 310009, China

^4^Transvascular Implantation Devices Research Institute, Hangzhou, 310053，China

^5^Department of Cardiology, Tongji Hospital affiliated to Tongji University, Shanghai, China

^6^Department of Cardiology, The First Affiliated Hospital of Wenzhou Medical University, Wenzhou, China

^7^Department of Cardiology, The Second Affiliated Hospital of Wenzhou Medical University, Wenzhou, China

^*^Junyan Jin and Delong Chen contributed equally as first authors.

^†^Corresponding authors.

**Address for correspondence**:

**Jun Jiang**^†^, MD, PhD.

Department of Cardiology, Second Affiliated Hospital, Zhejiang University School of Medicine

88 Jiefang Road, Hangzhou 310009, China.

E-mail: jiang-jun@zju.edu.cn

**Jian'an Wang**^†^, MD, PhD

Department of Cardiology, The Second Affiliated Hospital of Zhejiang University School of Medicine

88 Jiefang Road, Hangzhou 310009, China

E-mail: wangjianan111@zju.edu.cn

**Running title:** Diagnostic Performance of a Novel OCT-FFR

**Word count**: 3139 words (excluding abstract, references, 3 Tables, and 5 Figures)

**Declarations**

**Data Availability Statement**

Patient-level data collected for this study will not be made publicly available. Any reasonable inquiries should be sent to the corresponding author.

**Funding Statement**

The study was funded by the National Science and Technology Major Project of the Ministry of Science and Technology of China (No. 2023ZD0504100), the National Natural Science Foundation of China (No. 82170332), and Transvascular Implantation Devices Research Institute (KY012025005).

**Conflict of Interest Disclosures**

All authors have reported that they have no relationships relevant to the contents of this paper to disclose.

**Ethics approval and consent to participate**

Our study was conducted in accordance with the Helsinki Declaration and was approved by the ethical review board of all participated centers.

**Permission to reproduce material from other sources**

Not applicable.

**Clinical trial registration**

URL: https://www.chictr.org.cn; Unique identifier: ChiCTR2500098052.

**Supplementary Material**

**Technical Appendix for OCT-FFR**

1. Data Acquisition and Input

The calculation of OCT-FFR requires the OCT raw data format (RAW), which is acquired via pullback imaging using the Conaris P80 and Mobile OCT (Vivolight, China) systems. Blood pressure information is inputted prior to the OCT pullback imaging and is saved within the raw data file. Data with poor OCT image quality that is difficult to analyze must be excluded. The OCT-FFR calculation is performed within the Vivolight OCT software analysis system.

2. OCT Image Lumen Segmentation and Area Calculation

We adopted UNet++ (Nested U-Net)[1] for OCT lumen segmentation. Built on an encoder–decoder architecture, UNet++ introduces dense nested skip connections between the encoder and decoder sub-networks, which effectively bridges the semantic gap between feature maps at different depths.

At each resolution level, feature maps are progressively refined through basic convolution blocks. Each node in the nested structure performs dense feature concatenation: it receives inputs from all previous nodes at the same resolution level and the upsampled features from the deeper layer. This design enables efficient multi-scale feature aggregation and accurate boundary delineation.

The basic convolution block consists of a 3×3 convolution layer, batch normalization (BN), and a ReLU activation function. Downsampling is implemented via 2×2 max pooling, while bilinear upsampling is employed to restore the feature map size. Furthermore, deep supervision is integrated by appending 1×1 convolution layers to the outputs of different levels, allowing for flexible model pruning and improved gradient flow.

3. OCT-FFR Calculation

OCT-FFR utilizes a fractional flow reserve calculation method based on a fluid dynamics model. This method predicts the impact of lumen area variations on the fractional flow reserve based on changes in the OCT lumen area and the fluid dynamics model. Specifically, it is calculated based on proximal and distal pressures, which can be expressed by the following formula [2][3]：

$\text{OCT}-\text{FFR}=\frac{P_{d}}{P_{a}}=\frac{P_{a}-\Delta P}{P_{a}},$ (1)

Where Pa represents the proximal pressure, which can be calculated from the systolic and diastolic blood pressures：

$$P_{a}=\frac{2}{3}\times\text{Diastolic Blood Pressure}+\frac{1}{3}\times\text{Systolic Blood Pressure}$$

Pd represents the distal pressure; and ΔP represents the pressure loss between the distal and proximal segments of the vessel. Gould et al. indicated in their study that the pressure loss ΔP across a stenosis can be described using the following simplified formula [2]:

$$\Delta P=FV+SV^{2}\text{，},$$

$\Delta P=\frac{8\pi\mu L}{A_{s}}\frac{A_{n}}{A_{s}}\times V+\frac{k\rho}{2}\left(\frac{A_{n}}{A_{s}}-1\right)^{2}\times V^{2},$ (2)

$$F=\frac{8\pi\mu L}{A_{s}}\frac{A_{n}}{A_{s}}, S=\frac{\rho}{2}\left(\frac{A_{n}}{A_{\text{MS}}}-1\right)^{2}.$$

Where μ represents the absolute blood viscosity, L represents the length of the stenosis, *A_n_* represents the cross-sectional area of the normal vessel (reference lumen area), *A_s_* represents the cross-sectional area of the stenotic segment, V represents the flow velocity, ρ represents the blood density, and k is a constant related to inlet and outlet effects, which is set to k=1 in this algorithm. F and S represent the pressure loss coefficients due to viscous friction and separation at the outlet, respectively. The absolute blood viscosity is μ=4.0×10^−3^Pa⋅s, and the blood density is ρ=1050 kg/m^3^. A_MS_ represents the lumen area at the location of maximal stenosis (MS). Therefore, the coefficient S is calculated using the average area of the proximal and distal reference frames along with the lumen area at the maximal stenosis location.

4. OCT-FFR Value Output

After the OCT-FFR calculation is completed, the software reconstructs an unfolded carpet map based on the OCT-FFR value at the location of each frame, where a color gradient from red to yellow to blue indicates increasing OCT-FFR values. Ultimately, the OCT-FFR value at the most distal location of the vessel is used for comparison with the invasive FFR.

References

[1] Zhou, Zongwei, et al. "Unet++: A nested u-net architecture for medical image segmentation." International workshop on deep learning in medical image analysis. Cham: Springer International Publishing, 2018.

[2] Gould KL, Kelley KO, Bolson EL. Experimental validation of quantitative coronary arteriography for determining pressure-flow characteristics of coronary stenosis. Circulation. 1982 Nov;66(5):930-7.

[3] Seike, Fumiyasu , et al. Intracoronary Optical Coherence Tomography-Derived Virtual Fractional Flow Reserve for the Assessment of Coronary Artery Disease. American Journal of Cardiology. 2017:S0002914917312894.

**Table S1. Subgroup analysis of diagnostic performance of OCT-FFR to identify fractional flow reserve ≤ 0.80**

|  | **OCT-FFR ≤0.80 in LAD lesions (n=139)** | **OCT-FFR ≤0.80 in non-LAD lesions (n=61)** | **P value** | **OCT-FFR ≤0.80 in complex lesions (n=117)** | **OCT-FFR ≤0.80 in non-complex lesions (n=83)** | **P value** |
| --- | --- | --- | --- | --- | --- | --- |
| Accuracy, % (95% CI) | 97.8(93.8-99.6) | 98.4(91.2-100) | 1.00 | 97.4(92.7-99.5) | 98.8(93.5-100) | 0.87 |
| Sensitivity, % (95% CI) | 95.5(84.5-99.4) | 66.7(9.4-99.2) | 0.45 | 92.1(78.6-98.3) | 100(66.4-100) | 0.91 |
| Specificity, % (95% CI) | 98.9(94.3-100) | 100(93.8-100) | 1.00 | 100(95.4-100) | 98.6(92.7-100) | 0.97 |
| PPV, % (95% CI) | 97.7(85.7-99.7) | 100(15.8-100) | 1.00 | 100(90.0-100) | 90.0 (56.2-98.4) | 0.50 |
| NPV, % (95% CI) | 97.9(92.4-99.5) | 98.3(92.1-99.7) | 1.00 | 96.3(89.9-98.7) | 100(95.1-100) | 0.29 |

Values are % (95% CI).

Abbreviations: OCT-FFR, optical coherence tomography derived fractional flow reserve;LAD, left anterior descending; CI, confidence interval; PPV, positive predictive value; NPV, negative predictive value.

**Table S2. Subgroup analysis of OCT-FFR diagnostic performance according to OCT-derived plaque morphologies**

|  | **Lipid-rich plaque,**  **Present (n=57)** | **Lipid-rich plaque,**  **Absent (n=143)** | **P value** | **Fibrous plaque,**  **Present (n=94)** | **Fibrous plaque,**  **Absent (n=106)** | **P value** | **Calcification, Present(n=49)** | **Calcification, Absent (n=151)** | **P value** |
| --- | --- | --- | --- | --- | --- | --- | --- | --- | --- |
| Accuracy, % (95% CI) | 98.2 (90.6-100.0) | 97.9 (94.0-99.6) | 1.00 | 96.8 (91.0-99.3) | 99.1 (94.9-100.0) | 0.34 | 100.0 (92.7-100.0) | 97.4 (93.4-99.3) | 0.57 |
| Sensitivity, % (95% CI) | 92.3 (64.0-99.8) | 94.1 (80.3-99.3) | 1.00 | 91.7 (73.0-99.0) | 95.7 (78.1-99.9) | 1.00 | 100.0 (71.5-100.0) | 91.7 (77.5-98.2) | 1.00 |
| Specificity, % (95% CI) | 100.0 (92.0-100.0) | 99.1 (95.0-100.0) | 1.00 | 98.6 (92.3-100.0) | 100.0 (95.7-100.0) | 0.46 | 100.0 (90.7-100.0) | 99.1 (95.3-100.0) | 1.00 |
| PPV,  % (95% CI) | 100.0 (73.5-100.0) | 97.0 (84.2-99.9) | 1.00 | 95.7 (78.1-99.9) | 100.0 (84.6-100.0) | 1.00 | 100.0 (71.5-100.0) | 97.1 (84.7-99.9) | 1.00 |
| NPV,  % (95% CI) | 97.8 (88.2-99.9) | 98.2 (93.6-99.8) | 1.00 | 97.2 (90.2-99.7) | 98.8 (93.5-100.0) | 0.59 | 100.0 (90.7-100.0) | 97.4 (92.7-99.5) | 1.00 |

Values are % (95% CI).

Abbreviations: OCT-FFR, optical coherence tomography derived fractional flow reserve; CI, confidence interval; PPV, positive predictive value; NPV, negative predictive value.

**Table S3. Subgroup analysis of OCT-FFR diagnostic performance according to high-risk populations**

|  | **Diabetes mellitus (n=57)** | **Non-diabetic (n=143)** | **P value** | **Hypertension (n=94)** | **Non-hypertensive (n=106)** | **P value** | **Smoker**  **(n=49)** | **Non-smoker (n=151)** | **P value** |
| --- | --- | --- | --- | --- | --- | --- | --- | --- | --- |
| Accuracy, % (95% CI) | 96.9 (89.3-99.6) | 98.5 (94.8-99.8) | 0.60 | 98.3 (94.0-99.8) | 97.6 (91.5-99.7) | 1.00 | 98.0 (89.4-99.9) | 98.0 (94.2-99.6) | 1.00 |
| Sensitivity, % (95% CI) | 86.7 (59.5-98.3) | 96.9 (83.8-99.9) | 0.24 | 92.9 (76.5-99.1) | 94.7 (74.0-99.9) | 1.00 | 91.7 (61.5-99.8) | 94.1 (80.3-99.3) | 1.00 |
| Specificity, % (95% CI) | 100.0 (92.9-100.0) | 99.0 (94.7-100.0) | 1.00 | 100.0 (96.0-100.0) | 98.4 (91.5-100.0) | 0.41 | 100.0 (90.7-100.0) | 99.1 (95.2-100.0) | 1.00 |
| PPV,  % (95% CI) | 100.0 (75.3-100.0) | 96.9 (83.8-99.9) | 1.00 | 100.0 (86.8-100.0) | 94.7 (74.0-99.9) | 0.42 | 100.0 (71.5-100.0) | 97.0 (84.2-99.9) | 1.00 |
| NPV,  % (95% CI) | 96.2 (86.8-99.5) | 99.0 (94.7-100.0) | 0.26 | 97.8 (92.4-99.7) | 98.4 (91.5-100.0) | 1.00 | 97.4 (86.5-99.9) | 98.3 (93.9-99.8) | 1.00 |

Values are % (95% CI).

Abbreviations: OCT-FFR, optical coherence tomography derived fractional flow reserve; CI, confidence interval; PPV, positive predictive value; NPV, negative predictive value.

Figure S1. Study flowchart. FFR measurements and OCT imaging were performed in the target coronary vessel in a randomized sequence. The notation “FFR‑OCT” indicates that FFR was performed first, followed by OCT, whereas “OCT‑FFR” indicates that OCT was performed first, followed by FFR.

FFR, fractional flow reserve; OCT-FFR, optical coherence tomography.
